# Camera-trapping: wild and domestic species occurrences in three Pyrenean pastures

**DOI:** 10.3897/BDJ.12.e126097

**Published:** 2024-06-11

**Authors:** Manon Culos, Alice Ouvrier, Emilie Lerigoleur, Sarah Bitsch, Marie Dewost, Anaïs Guédon, Jonas Guignet, Agathe Le Guével, Aymeric Metz, Oscar Vilbert, Coline Vinette, Ruppert Vimal

**Affiliations:** 1 Association Dissonances, Bonac-Irazein, France Association Dissonances Bonac-Irazein France; 2 UMR 5602 Géographie de l'Environnement (CNRS/Université Toulouse 2), Toulouse, France UMR 5602 Géographie de l'Environnement (CNRS/Université Toulouse 2) Toulouse France

**Keywords:** monitoring, wildlife, pastoralism, *Ursusarctos*, grazing lands, Ariège

## Abstract

**Background:**

The co-existence between brown bears (*Ursusarctos* Linnaeus, 1758) and farmers in the Pyrenees has been a major concern for several decades. The bear's depredation on livestock has multiple implications for traditional practices of extensive grazing and calls for a better understanding of the various ways in which humans and non-humans interact across different territories. The present dataset stems from "The Pastoralism and Bears in the Pyrenees" research project led by the GEODE laboratory (UMR 5602 CNRS-UT2J) in partnership with the Association Dissonances. Focusing on three summer pastures as places of encounter, this project proposes to explore the definition of co-existence, based on context-dependent and constantly evolving relationships between bears and pastoralists. As part of an interdisciplinary approach combining animal geography and ecology, the spatio-temporal activity of the different species was explored using a network of 118 camera traps.

**New information:**

The 118 camera traps were installed on the three summer pastures while livestock was present in the mountains between May and October, from 2021 to 2023 and were set in a 400 m ✕ 400 m grid covering a total area of around 2,000 ha. The present dataset contains 57,928 occurrences of 22 taxon categories, including 19 identified species, two family categories (equids and mustelids) and one class category (birds). As pastoral activity is significantly present in these areas, livestock (sheep (*Ovisaries* Linnaeus, 1758), equids, cows (*Bostaurus* Linnaeus, 1758) and goats (*Caprahircus* Linnaeus, 1758)) account for 16,207 occurrences across the three pastures. The three main wild species captured over the three years and three pastures were the red deer (*Cervuselaphus* Linnaeus, 1758; 9,517 occurrences), red fox (*Vulpesvulpes* Linnaeus, 1758; 9,400 occurrences) and wild boar (*Susscrofa* Linnaeus, 1758; 4,016 occurrences).

Data are aggregated at the grid scale. Nonetheless, the exact locations of each camera trap as well as the photos can be requested from us.

## Introduction

Pyrenees combine both a strong cultural identity and a rich and endemic biodiversity (Gibon et al. 2004, Chandivert 2016, Eychenne 2018). Practised for centuries and still predominant in most areas, agropastoralism has played a key role in shaping today’s ecosystems (Davasse et al. 1996, Gibon and Balent 2005, Rendu et al. 2016).

For almost three decades, however, the return of the brown bear (*Ursusarctos*) following its re-introduction in 1996 has challenged the co-existence between wildlife and human activities. In particular, the summer pastures have become places of new encounters (Barua 2016), in which farmers and their cattle have had to adapt to the presence of bears.

Focusing on three of those pastures, the project “Pastoralism and Bears in the Pyrenees” aims to understand how humans and non-humans learn to share a common territory. Grounded in the burgeoning field of animal geography, it is attached to describe multispecies landscapes as places shaped by different animals with their specific histories and spatialities (Hodgetts and Lorimer 2014, Aisher and Damodaran 2016). In such approaches, understanding the multiple pathways through which humans and non-humans are intertwined is meant to open new opportunities for place-based, context-dependent conservation (Williams et al. 2013).

Amongst the various multi-disciplinary and complementary approaches that have been implemented to meet these objectives, a network of 118 camera traps distributed on the three pastures was central to tracking various species’ spatialities. It allowed us to understand how bears and sheep, as well as other species, spatially interact, how this changes through time and from one specific place to another.

## Project description

### Title

Camera-trapping: wild and domestic species occurrences in three Pyrenean pastures.

### Personnel

Alice Ouvrier, Ruppert Vimal, Anaïs Guédon, Agathe Le Guével, Aymeric Metz, Sarah Bitsch, Manon Culos, Marie Dewost, Coline Vinette, Oscar Vilbert, Jonas Guignet.

### Study area description

The study was conducted in three Pyrenean summer pastures, Arreau, Barestet and Ourdouas, two of which are located in the Ariège District and one of which is located in both the districts of Ariège and of Haute-Garonne, France. The three pastures are mainly represented by open habitats with vegetation composed of short grassy lawns and heaths with rhododendron (*Rhododendronferrugineum* L., 1753), calluna (*Callunavulgaris* (L.) Hull, 1808), blueberry (*Vacciniummyrtillus* L., 1753) and juniper (*Juniperuscommunis* L., 1753), but also present are variable surfaces of deciduous and coniferous forests (e.g. beech-fir forests). The pastures are grazed by livestock from May/June to October, following a grazing rotation system.

The pastures are all located within the Ariège Pyrenees Regional Nature Park and are also all located wholly or partly within Natura 2000 areas. The Barestet pasture is partly located in the ZSC (Special Area of Conservation) and ZPS (Special Protection Area) of the "Haute Vallée de la Garonne". The Ourdouas pasture is located in the ZSC and ZPS of "Vallée de l'Isard, Mail de Bulard, Pic de Maubermé, Pic de Serre-Haute and Pic du Crabère". The Arreau pasture is located in the ZPS "Massif du Mont Valier" and partly in the ZSC "Vallée du Riberot and Massif du Mont Valier". The Arreau pasture is also located in the Mont Valier State Reserve.

The Arreau pasture is located in the Municipality of Seix. It is the largest pasture since it reaches nearly 890 ha and ranges from 1,350 m to 2,470 m of elevation. The relief is varied, with rocky bars, scree and sometimes very steep slopes, but also streams with variable flow, a permanent pond and other fluctuating water surfaces are present. The anthropogenic frequentation of the pasture is divided into two parts: i) the pastoral activity of the site with the grazing of about 1,900 sheep and around 100 cows, horses (*Equuscaballus* Linnaeus, 1758) and donkeys (*Equusasinus* Linnaeus, 1758) and ii) the touristic activity favoured by the track crossing the pasture from the Col de Pause to the Port d'Aula and by the presence of cabins and the GR10 (i.e. highly frequented hiking trail). Pastoral activity in the pasture also includes six herding dogs (*Canisfamiliaris* Linnaeus, 1758) and three guarding dogs.

The Barestet pasture, located on the communes of Melles (Haute-Garonne) and Saint-Lary (Ariège), occupies a surface of 670 ha and ranges from 1,100 m to 2,150 m of elevation. This pasture is elongated, following a ridge linking the Cap de Gauch in the south, the highest point of the pasture, to the Puech in the north. Due to its accessibility and its hiking trails, part of the pasture is visited by a relatively large number of users, including tourists and hunters, with, for example, the occasional organisation of trail races. In addition to this frequentation, the pasture is used for pastoral activity, including the grazing of about 800 sheep, around 60 horses and donkeys and five goats. Pastoral activity in the pasture also includes two herding dogs and no guarding dogs at all.

The Ourdouas pasture is located in the Municipality of Sentein, occupies a surface area of 527 ha and ranges from 1,050 m to 2,420 m of elevation, whose highest point is the Pic de l'Har. The anthropogenic frequentation of the pasture is mainly due to the grazing of about 700 sheep, 25 cows and around 10 horses and donkeys. In addition, hunting activities take place in the pasture and tourist frequentation is quite reduced, which might be because of the absence of large hiking trails. Pastoral activity in the pasture also includes two herding dogs and five guarding dogs.

See Table 1 below for a summary of pasture characteristics.

### Funding

Centre national de la recherche scientifique (CNRS), Direction régionale de l'Environnement, de l'Aménagement et du Logement (DREAL Occitanie), Fondation François Sommer, Office Français de la Biodiversité (OFB).

## Sampling methods

### Study extent

The present dataset was collected during three sampling campaigns carried out between May and October 2021, 2022 and 2023 on three mountain pastures located in the Ariège Pyrenees.

A total of 118 motion-triggered infrared cameras (Reconyx^®^ Hyperfire 2) were set on the three mountain pastures. As a result of an excessive number of thefts during the three campaigns, one camera has been removed from the Barestet pasture and data from the three years has been removed from the dataset (dataset from 118 cameras instead of the 119 initially installed in 2021). Depending on the pastures and the years, according to the field constraints, the sampling started and ended at variable dates, but at least between 12 June and 6 October.

Insofar as the project is likely to continue, the present dataset may evolve, following the same protocol and supplemented by additional years of data on one or more pastures.

### Sampling description

A former exploratory study, carried out in 2020 on one of the three study sites, tested the implementation of camera traps following a regular grid. However, the characteristics of the mountain environment and the topographical diversity of the pastures resulted in camera traps being set up in locations that were particularly unfavourable for the passage of wildlife, pastoral activity and humans (e.g. extremely steep slopes). As a result, the sampling was conducted following a regular grid of 400 m ✕ 400 m cells, in which one camera was installed at a chosen location of potential passages of the mammalian fauna (e.g. path, trail, mountain pass, thinning of vegetation etc.), thus providing a fine spatial anchor on the activity of the species and maximising their detectability. This fine-scale grid was relevant to the study in order to have the greatest possible insight into the spatilities of the various wild and domestic species, given the technical and financial constraints involved. For the purpose of consistency, the same mesh size was used on each of the sites, resulting in different numbers of cameras for the three pastures depending on their respective size (Arreau: 53 cameras, Barestet: 36 cameras, Ourdouas: 29 cameras).

Depending on the vegetation and the configuration of the site, the cameras were installed on a tree or on a stake at a height varying from 50 cm to 1 m from the ground, at approximately 3 m and perpendicular to the potential path of the animal. Cameras were set to take three photos without delay between consecutive triggers in burst mode, as long as motion was detected. Cameras automatically record the date, time, moon phase, temperature of the captured events and the unique station’s label. Further configuration details can be found in Table 2.

Each camera was checked approximately every three weeks for data recovery, battery replacement and camera maintenance. Despite regular and frequent monitoring of the camera traps throughout the study period, some traps did not operate continuously over the study period due to various technical problems, thefts or damage by cattle. Although the sampling effort of each camera, in days, is indicated in the dataset (e.g. 134 days), the details of the functioning days of each camera for each pasture can be found on the following DOI: https://doi.org/10.48579/PRO/XUSK8V, in order to locate data gaps over time and distinguish them from periods without capturing events. During the entire study period, a total of 49,271 trap-days were sampled. See details in Table 3.

The obtained pictures were manually identified on Timelapse Software.

For wild species, two pictures separated by a time interval of at least two minutes were considered different capture events. When a capture event was recorded, only the first photo where the species was identifiable was annotated (i.e. species, temperature, date, time and number of individuals). When different individuals of the same species appeared later in the same capture event, the total number of individuals in the capture event was filled in using the first annotated photo. However, if an individual of another species appeared later in the same capture event, a new photo was annotated as the first identifiable photo of the species. Consequently, the date and time indicated for each capture event do not necessarily correspond to the exact number of individuals passing through at that precise time, but rather to the start of an event of variable duration containing that number of individuals.

In some rare cases, two photos separated by an interval of more than two minutes were considered a single capture event. For example, an individual may have been sleeping in front of the camera, triggering it only occasionally. In such cases, a single event was recorded.

Regarding livestock (i.e. cows, equids, goats and sheep), the photos were annotated on the basis of an hour's time. Thus, the minimum time interval between two consecutive capture events was one hour. In addition, as their behaviour can involve long periods of static in front of the cameras, in cases where a domestic species was continuously recorded for several hours, a new capture event was registered every hour after the start of the event. Moreover, for livestock data, the number of individuals was not recorded precisely since it mainly corresponded to the passage of herds. Thus, the value entered in the dataset is “many.” A precise number of individuals was only registered when an event showed only five sheep or less in order to identify isolated groups of sheep, which was relevant to answering the study goals.

The dataset provided contains rows corresponding to each occurrence and, therefore, only shows information corresponding to the single annotated photo in each capture event. When two species appeared at the same time in a photo, the photo was annotated with both species, inducing two occurrences for one capture event. In the dataset, the row for this capture event was duplicated to obtain one row per occurrence.

### Quality control

The species identification of all tagged photos has been double-checked by the authors. At the first identification, the error rate was 0.32% and these identified errors were corrected at the second verification, bringing the final error rate to nearly 0%. Potential gaps (e.g. missing temperature information) have also been checked and photos of humans were systematically removed from the dataset.

### Step description

The study areas – three mountain pastures in Ariège – were selected on the basis of criteria relevant to the project (e.g. differences in pastoral activity, confirmed and dense bear presence, accessibility etc.). The choice of sectors was also dictated by the agreement of the pastoral groups present on the pastures to host the project for at least three years.

Authorisations to use vehicles on the tracks approaching the pastures and authorisations to set photographic traps were then requested from the manager of these territories, in this case the Office national des forêts (ONF), which oversees the integrity of the forest estate, the conservation of structures and the protection of forest stands and natural environments, as well as the management of wildlife and hunting.

A 400 m ✕ 400 m grid covering the entire surface of each mountain pasture was chosen according to the objectives of the study, as mentionned above and the location of the camera traps in each grid cell was chosen according to the most suitable place for fauna to pass (e.g. path, trail, mountain pass, thinning of vegetation etc.). When the camera traps were installed, the main habitat in which the cameras were installed was noted. As this information was not necessary for the project's objectives, habitat characterisation was done on sight and in an arbitrary manner. The cameras, set to take three photos without delay between consecutive triggers in burst mode as long as motion was detected, were installed approximately between May and October of the three years of the project, depending on field constraints (e.g. snow cover) and were checked approximately every three weeks.

The data collected were processed using Timelapse software, according to the photo completion protocol explained above. Photos have been identified at the class, family or species level and photos of humans were systematically removed from the dataset. The dataset was then checked to control identification errors and potential gaps (e.g. missing temperature information) and to standardise the data.

The dataset, deposited on the Global Biodiversity Information Facility platform (GBIF), was standardised in Darwin Core Archive (DwC-A) format to create an occurrence dataset corresponding to the standards recommended by Darwin Core.

## Geographic coverage

### Description

The present study focuses on three Pyrenean summer pastures, Arreau (around 890 ha), Barestet (around 645 ha) and Ourdouas (around 527 ha), two of which are located in the Ariège Department and one of which is located in both the Departments of Ariège and Haute-Garonne, France. The three pastures are respectively part of one, two and one municipalities: Seix, Melles – Saint-Lary and Sentein.

**Figure 1.**
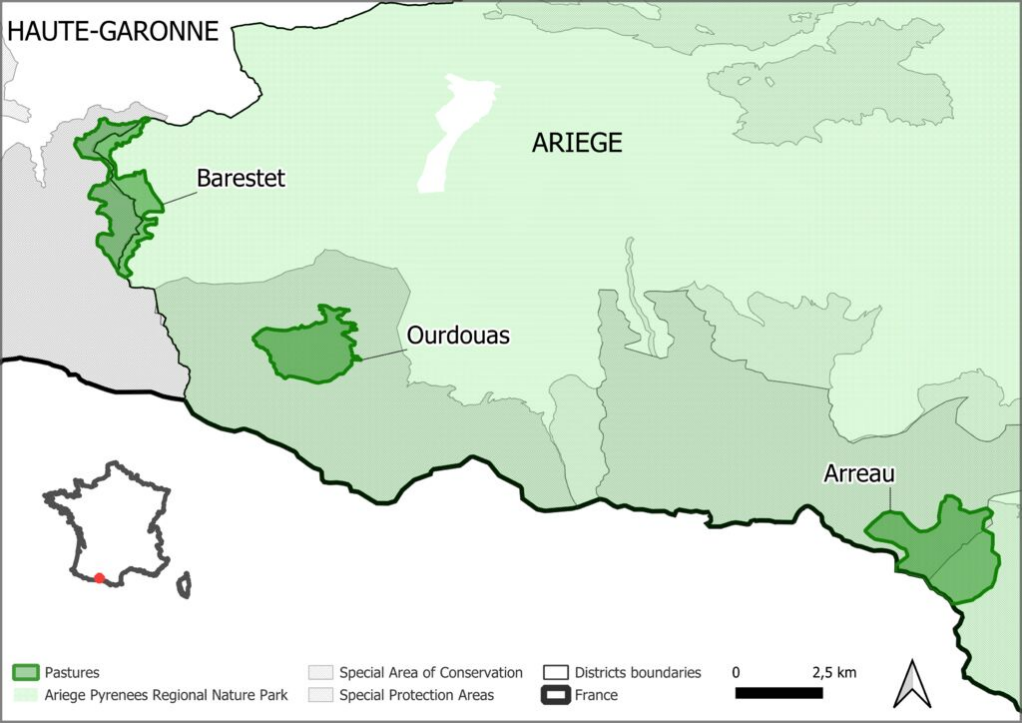
Location of the three mountain pastures.

The coordinates of each pasture's extent can be found in Table 4 and locations can be found below (Fig. 1).

### Coordinates

42.75484 and 42.92366 Latitude; 0.82063 and 1.14785 Longitude.

## Taxonomic coverage

### Description

Whenever possible, the taxonomic identification was made at the species level. Due to study objectives and identification difficulties, birds, equids and mustelids were not identified at the species level, but at the class level or at the family level, except for Western capercaillie and European badger. Thus, a total of one class, two families and nineteen species were recorded.

### Taxa included

**Table:** 

Rank	Scientific Name	Common Name
class	Aves	Birds
family	Equidae	Equids
family	Mustelidae	Mustelids
species	*Bostaurus* Linnaeus, 1758	Cow
species	*Canisfamiliaris* Linnaeus, 1758	Dog
species	*Caprahircus* Linnaeus, 1758	Feral goat
species	*Capreoluscapreolus* Linnaeus, 1758	Roe deer
species	*Cervuselaphus* Linnaeus, 1758	Red deer
species	*Damadama* Linnaeus, 1758	Fallow deer
species	*Erinaceuseuropaeus* Linnaeus, 1758	West european hedgehog
species	*Felissilvestris* Schreber, 1775	Wildcat
species	*Genettagenetta* Linnaeus, 1758	Common genet
species	*Lepuseuropaeus* Pallas, 1778	European hare
species	*Marmotamarmota* Linnaeus, 1758	Alpine marmot
species	*Melesmeles* Linnaeus, 1758	European badger
species	*Ovisaries* Linnaeus, 1758	Sheep
species	*Rupicaprapyrenaica* Bonaparte, 1845	Pyrenean chamois
species	*Sciurusvulgaris* Linnaeus, 1758	Eurasian red squirrel
species	*Susscrofa* Linnaeus, 1758	Wild boar
species	*Tetraourogallus* Linnaeus, 1758	Western capercaillie
species	*Ursusarctos* Linnaeus, 1758	Brown bear
species	*Vulpesvulpes* Linnaeus, 1758	Red fox

## Temporal coverage

**Data range:** 2021-5-12 – 2021-10-20; 2022-5-02 – 2022-10-23; 2023-5-03 – 2023-10-23.

## Usage licence

### Usage licence

Other

### IP rights notes

Creative Commons Attribution Non Commercial (CC-BY-NC 4.0)

## Data resources

### Data package title

Camera-trapping: wild and domestic species occurrences in three Pyrenean pastures

### Resource link

https://doi.org/10.15468/939z6d

### Alternative identifiers

https://ipt.gbif.fr/resource?r=pop_project_2021

### Number of data sets

1

### Data set 1.

#### Data set name

pop_project_2021

#### Data format

Darwin Core Archive

#### Description

This dataset is available on the Global Biodiversity Information Facility platform (GBIF) (Vimal et al. 2024) and contains 57,928 occurrences recorded at the class, family or species level. The species were identified following TaxRef 16.0, the national taxonomic reference system for the fauna, flora and fungi of mainland France and overseas, developed by the National Museum of Natural History. As part of the GBIF resource, this dataset is registered in the format corresponding to occurrence data. In addition, the dataset has been formatted according to the recommendations of the Darwin Core Archive (DwC-A), a tool facilitating the standardised publication of biodiversity data. The Darwin Core Standard (DwC) was used to structure occurrence data. It offers a stable, straightforward and flexible framework for compiling biodiversity occurrence data (Wieczorek et al. 2012). The table below describes the data dictionary with column labels corresponding to DwC descriptors, sometimes adjusted to match the data. The inscription eMoF stands for extended measurement or fact extension, which provides information about the air temperature measured. Moreover, this Darwin Core Archive includes standardised metadata written in XML format, using the open-source EML standard (Ecological Metadata Language).

In this dataset, each row corresponds to an occurrence. As an occurrence is part of a capture event composed of several photographs of the individual, each row corresponds to the single annotated photo in each capture event. When two species appeared at the same time in a photo, the photo was annotated with both species, resulting in two occurrences for one capture event. In the dataset, the row for this capture event was duplicated to obtain one row per occurrence.

With a view to preserving areas of quietness, life and passage for sensitive species, the GPS locations of brown bear and capercaillie (*Tetraourogallus* Linnaeus, 1758) occurrences, respectively classified as critically endangered and vulnerable on the French Red List, have been blurred. The coordinates shown correspond to the centroids of each summer pasture. For all other species, the location of each passage is given at the scale of the 400 m ✕ 400 m grid cell in which the camera trap that recorded the passage is installed.

**Data set 1.** 

Column label	Column description
occurrenceID	Unique identifier of the record.
basisOfRecord	The specific nature of the data record.
samplingProtocol	Description of the sampling method used.
samplingEffort	Number of days each camera trap was in operation.
decimalLongitude	The geographic longitude at which the occurrence took place.
decimalLatitude	The geographic longitude at which the occurrence took place.
footprintWKT	Either grid cell centroid, or pasture centroid, as specified in dataGeneralisations.
coordinateUncertaintyInMetres	Accuracy of coordinates given in decimalLongitude and decimalLatitude.
geodeticDatum	Spatial reference system (SRS) upon which the geographic coordinates given in footprintWKT are based.
dataGeneralisations	Details on the GPS coordinates indicated in footprintWKT.
country	Country of the sampling site.
countryCode	ISO code of the country.
eventDate	Date and time when the event occurred.
scientificName	The full scientific name, with authorship and date information, if known.
kingdom	The full scientific name of the kingdom in which the taxon is classified.
phylum	The full scientific name of the phylum or division in which the taxon is classified.
class	The full scientific name of the class in which the taxon is classified.
order	The full scientific name of the order in which the taxon is classified.
family	The full scientific name of the family in which the taxon is classified.
taxonRank	The taxonomic rank of the most specific name in the scientificName.
nameAccordingTo	The reference to the source in which the specific taxon concept circumscription is defined or implied.
dynamicProperties	French IUCN categories of each identified species.
occurrenceStatus	A statement about the presence or absence of a taxon at a location.
establishmentMeans	Statement about whether the species has been introduced in France through the direct or indirect activity of modern humans.
degreeOfEstablishment	The degree to which the species survives, reproduces and expands its range at the French level.
organismQuantity	A number or enumeration value for the quantity of organisms.
organismQuantityType	The type of quantification system used for the quantity of organisms.
habitat	Visual description of the main habitat in which the event occurred.
measurementID (eMoF)	An identifier for the MeasurementOrFact.
measurementType (eMoF)	The nature of the measurement, fact, characteristic or assertion.
measurementValue (eMoF)	The value of the measurement, fact, characteristic or assertion.
measurementUnit (eMoF)	The units associated with the measurementValue.

## Additional information

### Results

The 118 camera traps produced a total of 57,928 occurrences over the three years and the three pastures. This dataset includes the passages of two classes (birds and mammals), 13 families and 19 species (see Table 5).

As the identification of birds was not relevant to the study, only capercaillie were identified to species level. The other 2,493 occurrences were assigned to the "birds" category. Similarly, equids (in this case, horses and donkeys) were identified in the "equid" family only, with a total of 4,566 occurrences. Finally, within the mustelid family, only badgers (*Melesmeles* Linnaeus, 1758) were identified to the species level. The other 544 occurrences were recorded in the 'mustelids' category.

In total, the dataset comprises 22 taxon categories, including 19 identified species, two family categories (equids and mustelids) and one class category (birds).

At the level of the three mountain pastures, the wild species with the most occurrences is the red deer (9,517 occurrences), followed by the red fox (9,400 occurrences) and the wild boar (4,016 occurrences). As pastoral activity is significantly present in these areas, livestock accounts for a large proportion of the occurrences produced across the three pastures, with 16,207 occurrences: sheep (9,274 occurrences), equids (4,566 occurrences), cows (2,289 occurrences) and goats (78 occurrences).

The pastures are distinguished from one another by the presence or absence of certain species, as well as by their capture rate (i.e. number of independent records weighted by the number of trap-days).

The Arreau pasture, with 53 camera traps and 19,546 trap-days in total, stands out from the other pastures for its presence of marmots (*Marmotamarmota* Linnaeus, 1758; 774 occurrences in total), a low presence of red deer (134 occurrences) and mustelids (94 occurrences).

The Barestet pasture, with 36 photographic traps and 16,923 trap-days, was characterised by the capture events of a hedgehog (*Erinaceuseuropaeus* Linnaeus, 1758), a genet (*Genettagenetta* Linnaeus, 1758) and fallow deer (*Damadama* Linnaeus, 1758) in 2023. This pasture also has a large number of occurrences of red deer (6,415 occurrences).

The Ourdouas pasture, with 29 photographic traps and 12,802 trap-days, recorded 19 capercaillie occurrences.

In line with the objectives of the study, these data were supplemented by semi-structured interviews with members of pastoral groups and experts in charge of biodiversity monitoring programmes, as well as by institutional data providing information on pastoral activity and bears. All of these data have enabled us to highlight that the spatial use of the pastures by pastoralists (i.e shepherd, farmers, sheep) is confined within the boundaries of the pastures and follows a rotational grazing cycle for all the pasture areas. Conversely, the data suggest that bears use the mountain pastures in a more opportunistic and unpredictable way. In this context, taking into account the other species present on the pastures has revealed the specificities of the bears/farmers relationship. Overall, our results show how the spatial entanglement between humans and non-humans varies according to the study sites and the species considered. These results encourage the adoption of a place-based approach of co-existence.

### Data request

Insofar as the project is likely to continue, the present dataset may evolve, following the same protocol, being supplemented by additional years of data from one or more pastures.

**Figure 2.**
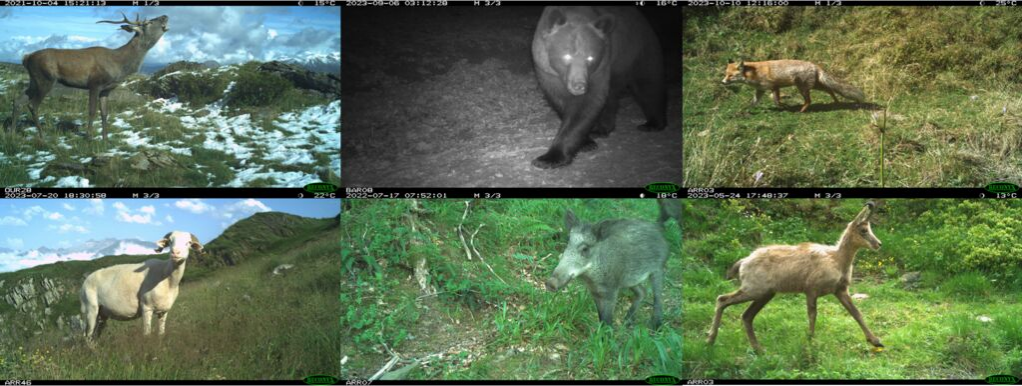
Picture of a red deer (Ourdouas), a brown bear (Barestet), a red fox, a sheep, a wild boar and a pyrenean chamois (Arreau).

Although the raw data (pictures) are not made available and since these data might be useful to some projects (e.g. species recognition software), it is possible to contact us to obtain the pictures, which are sorted by camera trap and by time period. See below for an example of pictures (Fig. 2).

In addition, as mentioned above, the exact coordinates of the camera traps are blurred, either at the scale of the 400 m ✕ 400 m grid cell centroid or at the scale of the pasture centroid. While the dataset therefore only shows points (two centroid scales), the geometry of each grid cell can be found in the additional files (https://doi.org/10.48579/PRO/XUSK8V) to facilitate the cartographic use of the data.

To work directly with the exact locations of brown bear, capercaillie and other species capture events, please contact us directly.

## Figures and Tables

**Table 1.** General characteristics of the pastures. Habitat percentages were calculated using QGIS 3.14 and based on the OSO 2021 land-cover layer (value-added data processed by CNES for the Theia data centre from Copernicus data; the processing uses algorithms developed by Theia's Scientific Expertise Centres).

Characteristic		Arreau	Barestet	Ourdouas
General characteristics	Elevation min (m)	1,350	1,100	1,050
	Elevation max (m)	2,470	2,150	2,420
	Surface (Ha)	890	670	527
Pastoral activity	Sheep herd size	1900	800	700
	Cow herd size	100	0	25
	Equid herd size		60	10
	Goat herd size	0	5	0
	Number of herding dogs	6	2	2
	Number of guarding dogs	3	0	5
Habitat	Heath proportion (%)	76	51	71
	Forest proportion (%)	3	37	19
	Grassy lawns proportion (%)	20	12	7

**Table 2.** Camera traps configuration parameters.

**Settings**	**Chosen option**
Motion pictures	On
Number of pictures	3
Time between pictures	No delay
Motion video	Off
Sensitivity	Medium/High
Day/Night operation	Both
Time format	24 Hr
Temperture format	°C
Location	Other
Resolution	72 ppp
Battery type	NiMH rechargeable batteries

**Table 3.** Number of trap-days for each year and each pasture.

Pasture	2021	2022	2023	Total
Arreau	5,742	6,777	7,027	19,546
Barestet	4,782	6,053	6,088	16,923
Ourdouas	4,011	4,351	4,440	12,802
Total	14,535	17,181	17,555	49,271

**Table 4.** Coordinates of each pasture’s extent.

	Arreau pasture		Barestet pasture		Ourdouas pasture	
	Longitude	Latitude	Longitude	Latitude	Longitude	Latitude
NW	1.09884	42.79262	0.82015	42.92595	0.88280	42.85976
NE	1.14628	42.79262	0.85128	42.92595	0.92123	42.85976
SE	1.14628	42.75455	0.85128	42.86946	0.92123	42.83244
SW	1.09884	42.75455	0.82015	42.86946	0.88280	42.83244

**Table 5.** Number of records and IUCN categories (NA: Not Applicable; NE: Not Evaluated; LC: Least Concern; VU Vulnerable; CR: Critically Endangered) of the 22 taxa recorded by camera trapping on the pastures of Arreau, Barestet and Ourdouas between 2021 and 2023.

Class	Family	Species	IUCN (FR)	IUCN (World)	Arreau (53 CT)	Barestet (36 CT)	Ourdouas (29 CT)	Total
Aves (Birds)	-	-	-	-	1336	759	398	2493
	Phasianidae	*Tetraourogallus* (Western capercaillie)	VU	LC	0	2	19	21
Mammalia	Bovidae	*Bostaurus* (Cow)	NE	NA	1170	10	1109	2289
		*Caprahircus* (Feral goat)	NE	NA	0	78	0	78
		*Ovisaries* (Sheep)	NE	NA	3487	3475	2312	9274
		*Rupicaprapyrenaica* (Pyrenean chamois)	LC	LC	391	358	183	932
	Canidae	*Canisfamiliaris* (Dog)	NE	NA	4926	2197	3061	10184
		*Vulpesvulpes* (Red fox)	LC	LC	4481	2314	2605	9400
	Cervidae	*Capreoluscapreolus* (Roe deer)	LC	LC	663	414	537	1614
		*Cervuselaphus* (Red deer)	LC	LC	134	6415	2968	9517
		*Damadama* (Fallow Deer)	NA	LC	0	6	0	6
	*Equidae*	-	-	-	1099	2681	786	4566
	Erinaceidae	*Erinaceuseuropaeus* (West European hedgehog)	LC	LC	0	1	0	1
	Felidae	*Felissilvestris* (Wildcat)	LC	LC	120	174	83	377
	Leporidae	*Lepuseuropaeus* (European hare)	LC	LC	2	914	7	923
	Mustelidae	-	-	-	68	381	95	544
		*Melesmeles* (European badger)	LC	LC	26	230	146	402
	Sciuridae	*Marmotamarmota* (Alpine marmot)	LC	LC	774	0	0	774
		*Sciurusvulgaris* (Eurasian red squirrel)	LC	LC	6	40	16	62
	Suidae	*Susscrofa* (Wild boar)	LC	LC	1332	1681	1003	4016
	Ursidae	*Ursusarctos* (Brown bear)	CR	LC	154	186	114	454
	Viverridae	*Genettagenetta* (Common genet)	LC	LC	0	1	0	1
